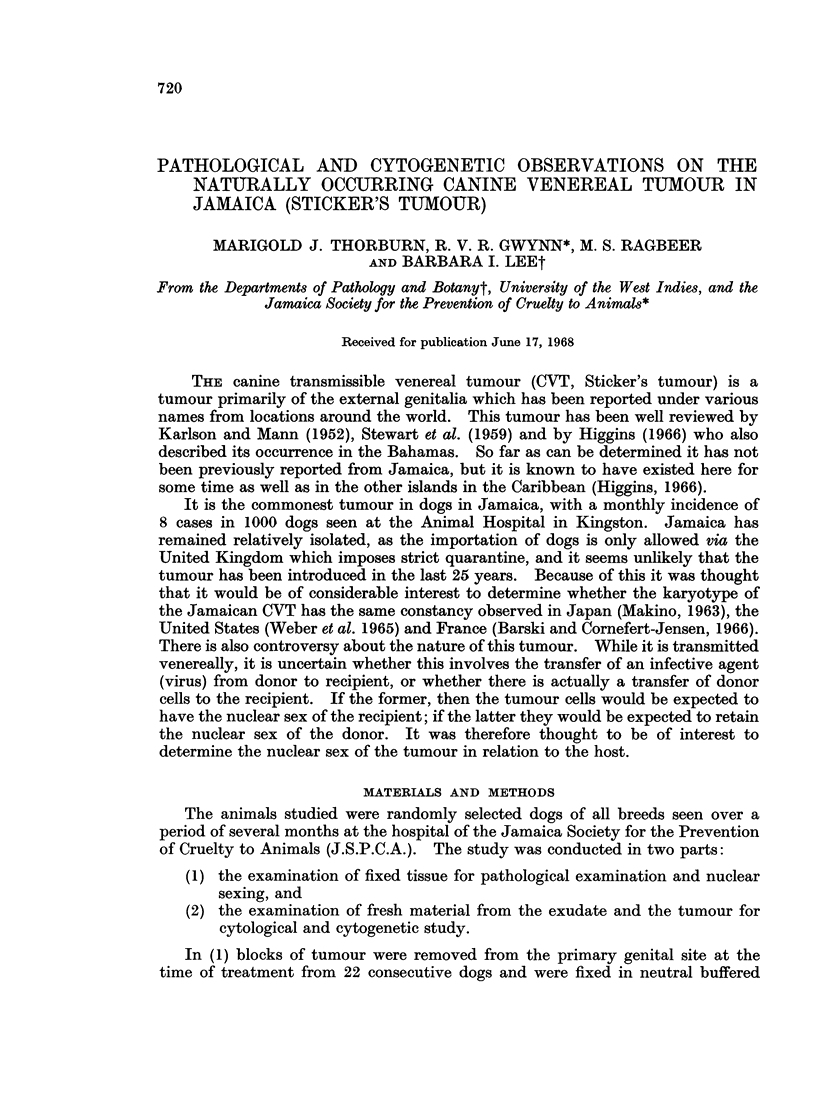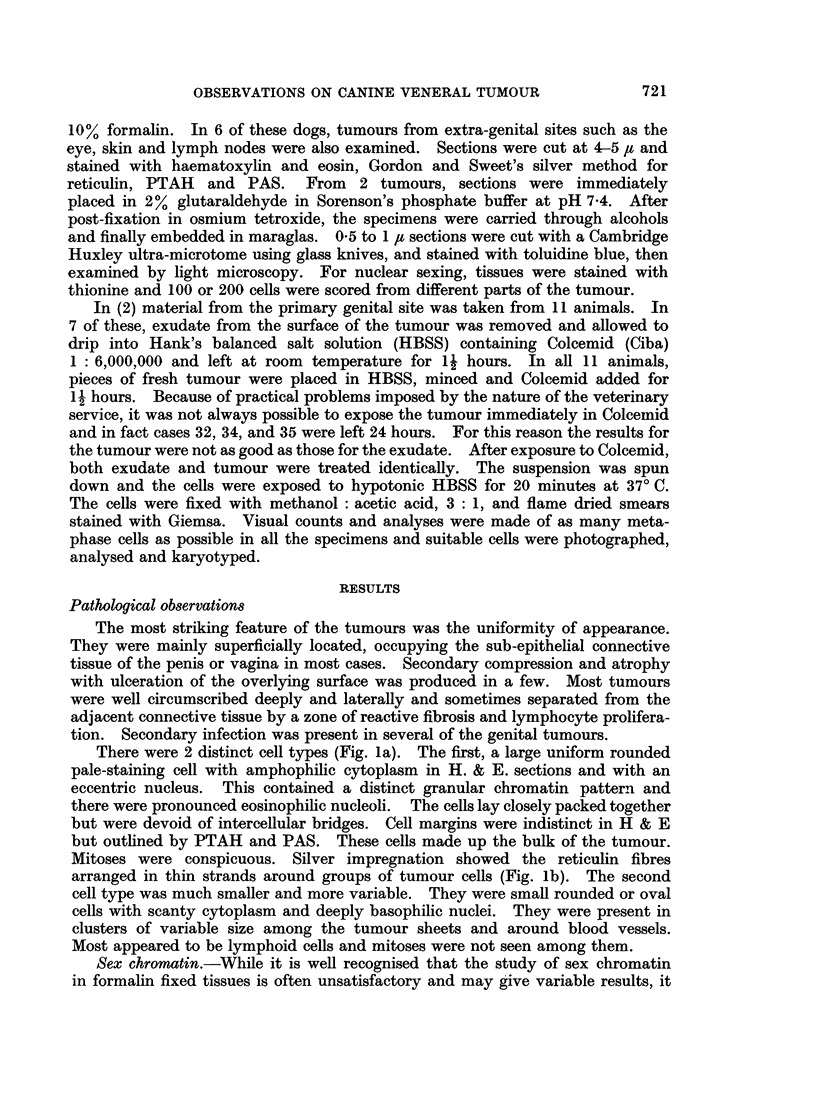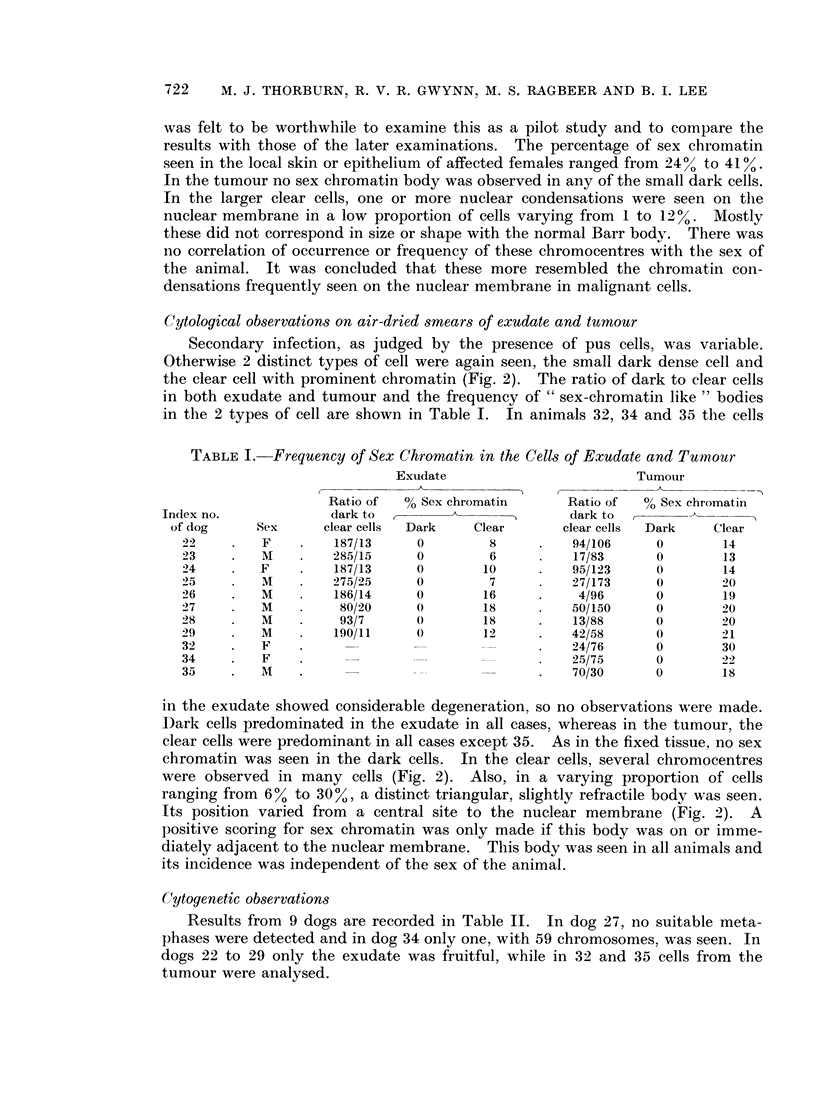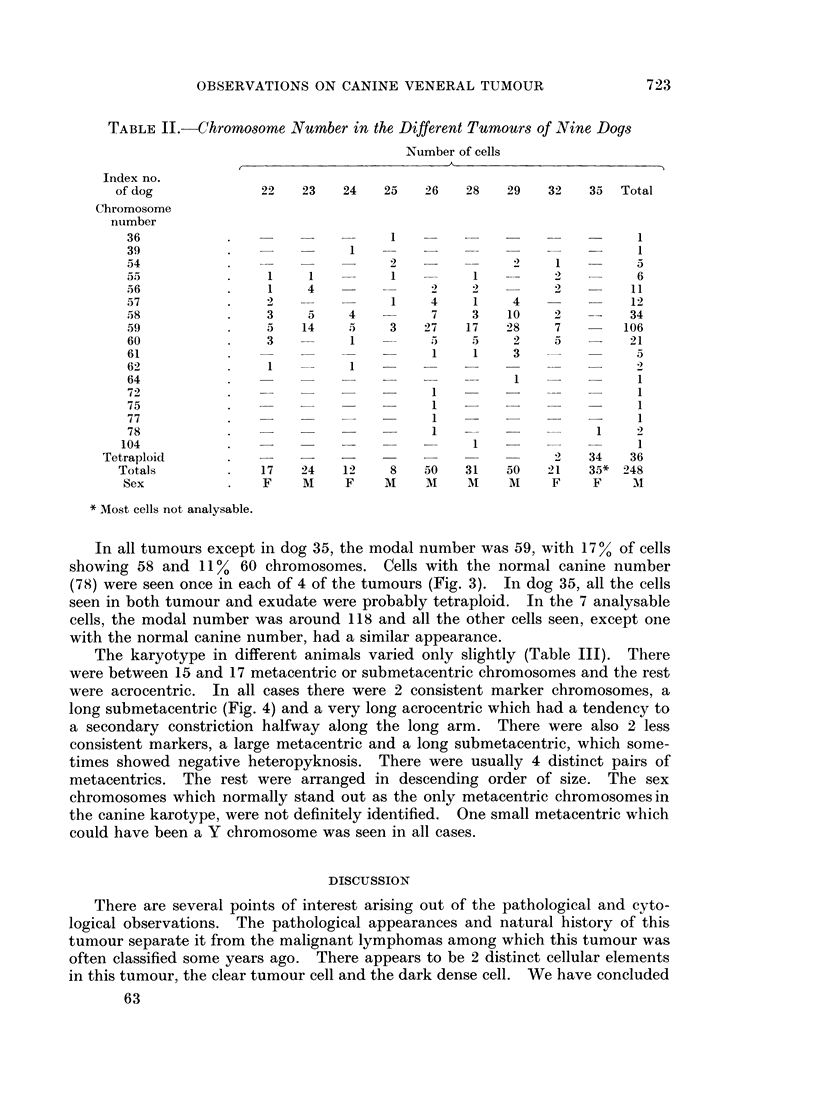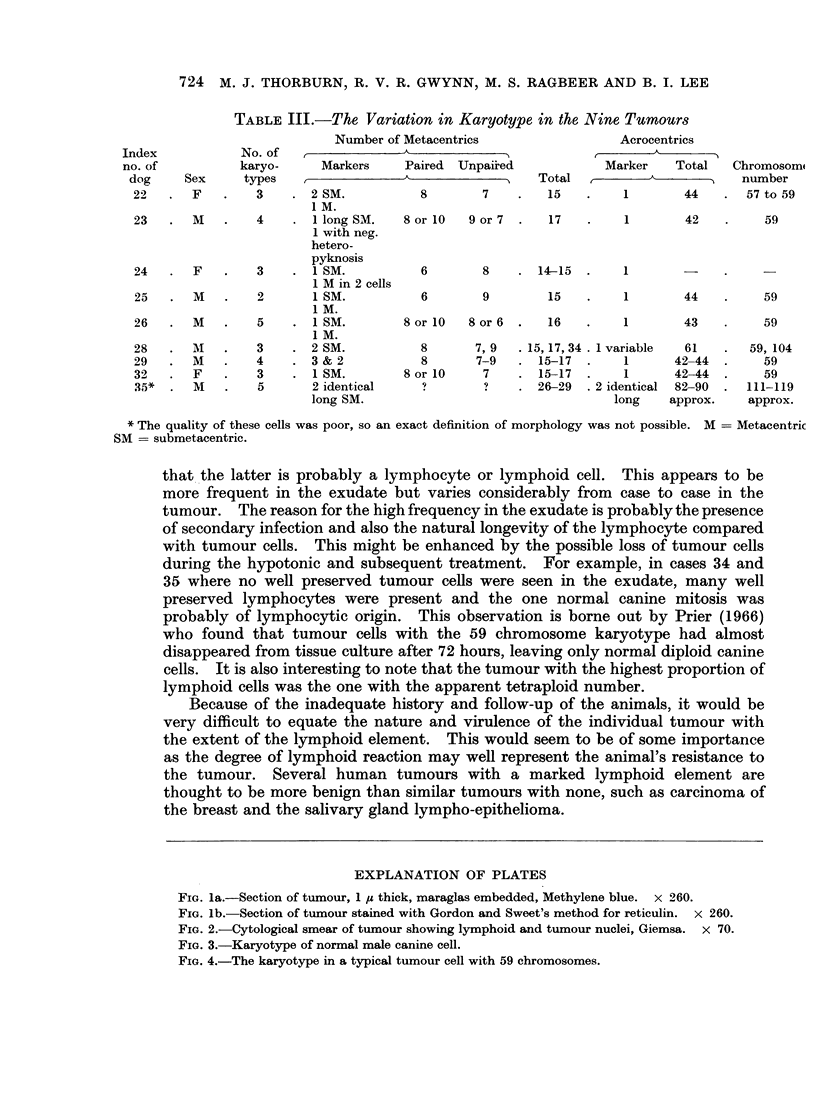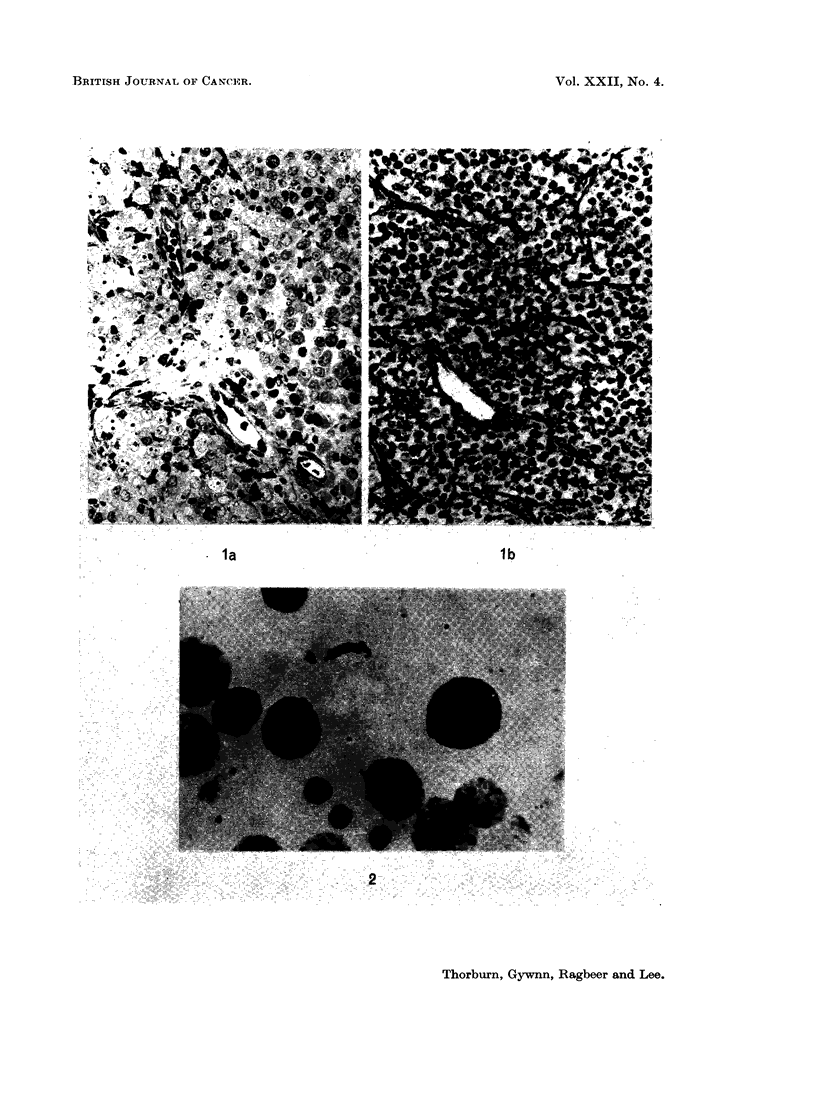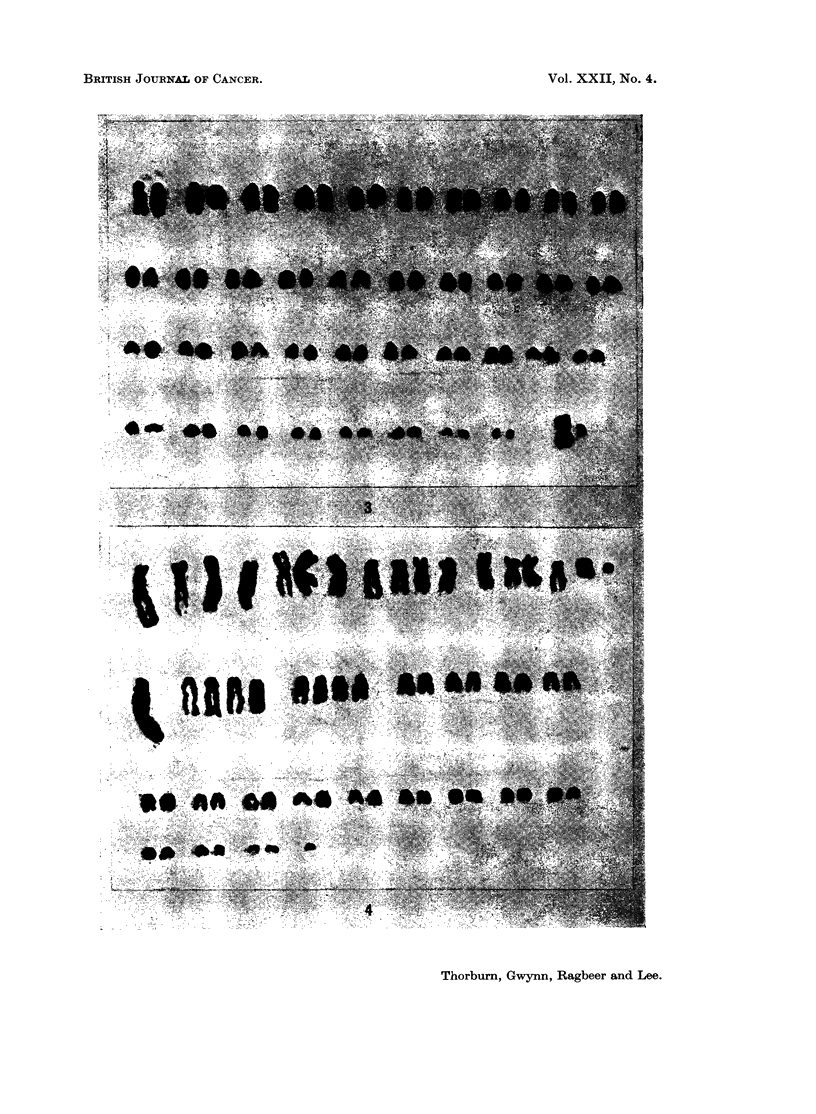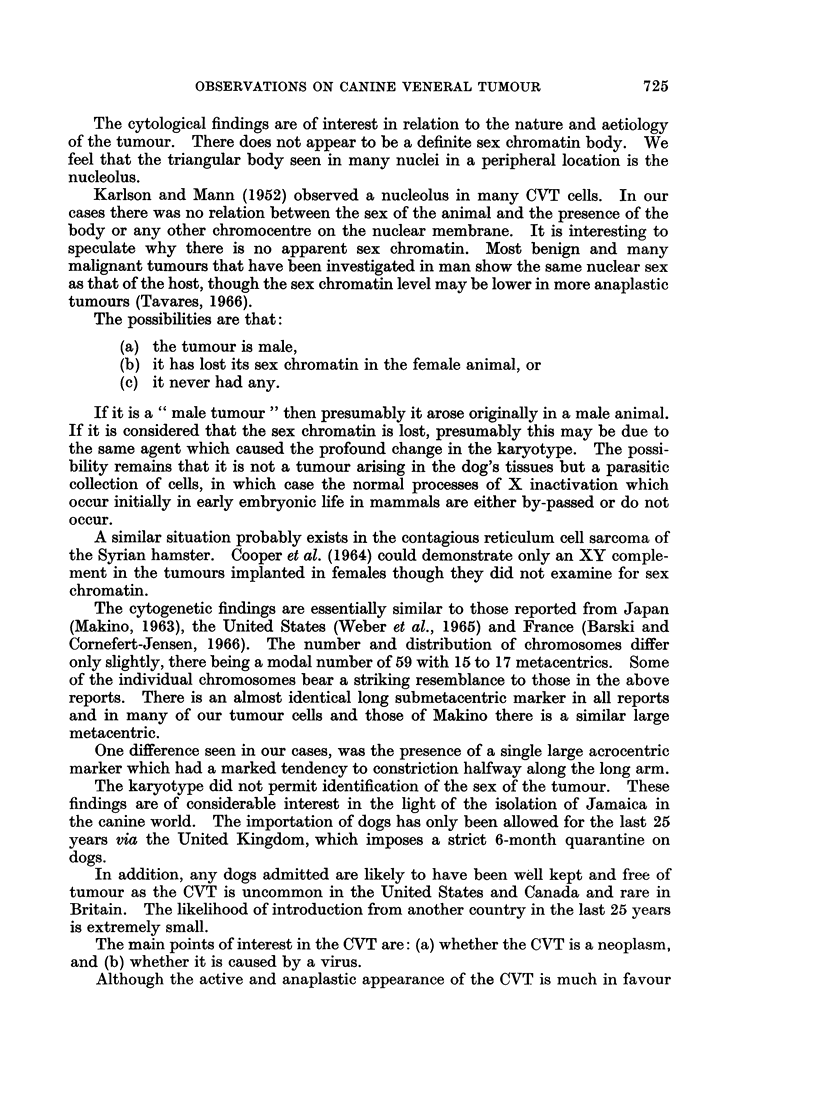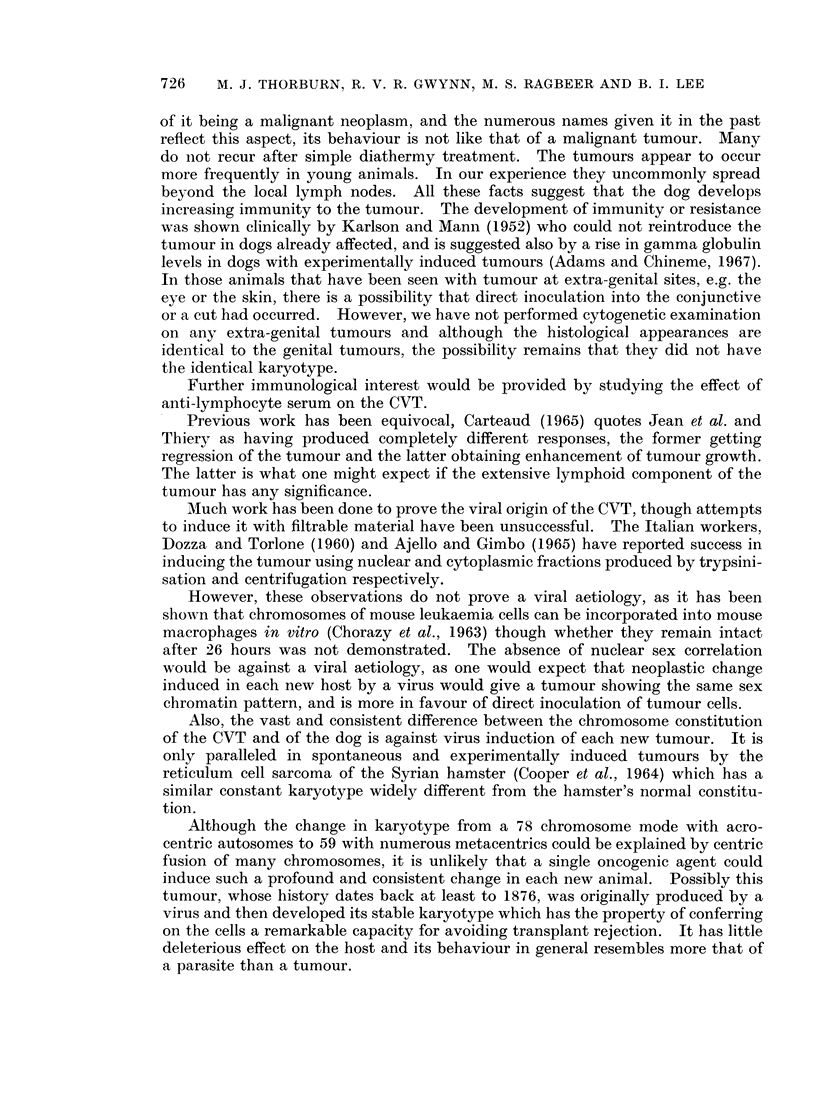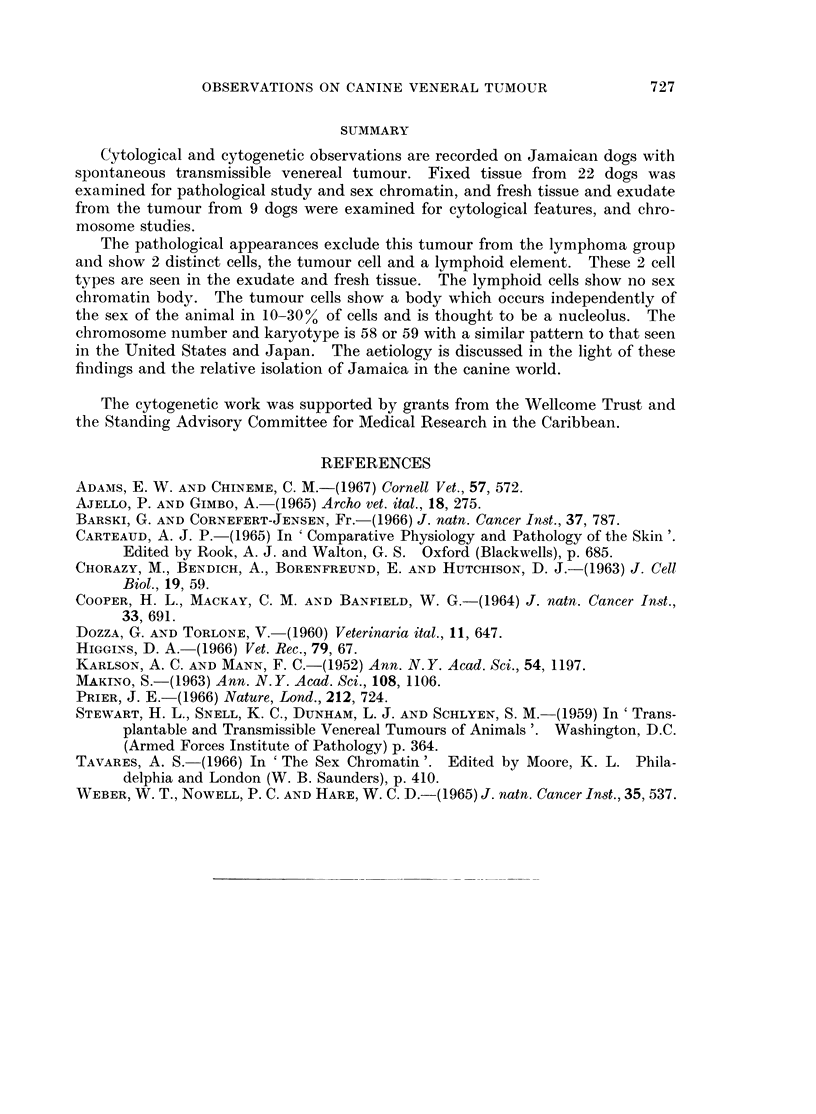# Pathological and cytogenetic observations on the naturally occurring canine venereal tumour in Jamaica (Sticker's tumour).

**DOI:** 10.1038/bjc.1968.86

**Published:** 1968-12

**Authors:** M. J. Thorburn, R. V. Gwynn, M. S. Ragbeer, B. I. Lee

## Abstract

**Images:**


### PATHOLOGICAL AND CYTOGENETIC OBSERVATIONS ON THE NATURALLY OCCURRING CANINE VENEREAL TUMOUR IN JAMAICA (STICKER'S TUMOUR)

MARIGOLD J. THORBURN, R. V. R. GWYNN*, M. S. RAGBEER AND BARBARA I. LEE†

*From the Departments of Pathology and Botany†, University of the West Indies, and the Jamaica Society for the Prevention of Cruelty to Animals**

Received for publication June 17, 1968

THE canine transmissible venereal tumour (CVT, Sticker's tumour) is a tumour primarily of the external genitalia which has been reported under various names from locations around the world. This tumour has been well reviewed by Karlson and Mann (1952), Stewart *et al.* (1959) and by Higgins (1966) who also described its occurrence in the Bahamas. So far as can be determined it has not been previously reported from Jamaica, but it is known to have existed here for some time as well as in the other islands in the Caribbean (Higgins, 1966).

It is the commonest tumour in dogs in Jamaica, with a monthly incidence of 8 cases in 1000 dogs seen at the Animal Hospital in Kingston. Jamaica has remained relatively isolated, as the importation of dogs is only allowed *via* the United Kingdom which imposes strict quarantine, and it seems unlikely that the tumour has been introduced in the last 25 years. Because of this it was thought that it would be of considerable interest to determine whether the karyotype of the Jamaican CVT has the same constancy observed in Japan (Makino, 1963), the United States (Weber *et al.* 1965) and France (Barski and Cornefert-Jensen, 1966). There is also controversy about the nature of this tumour. While it is transmitted venereally, it is uncertain whether this involves the transfer of an infective agent (virus) from donor to recipient, or whether there is actually a transfer of donor cells to the recipient. If the former, then the tumour cells would be expected to have the nuclear sex of the recipient; if the latter they would be expected to retain the nuclear sex of the donor. It was therefore thought to be of interest to determine the nuclear sex of the tumour in relation to the host.

### MATERIALS AND METHODS

The animals studied were randomly selected dogs of all breeds seen over a period of several months at the hospital of the Jamaica Society for the Prevention of Cruelty to Animals (J.S.P.C.A.). The study was conducted in two parts:

(1) the examination of fixed tissue for pathological examination and nuclear sexing, and
(2) the examination of fresh material from the exudate and the tumour for cytological and cytogenetic study.

In (1) blocks of tumour were removed from the primary genital site at the time of treatment from 22 consecutive dogs and were fixed in neutral buffered

10% formalin. In 6 of these dogs, tumours from extra-genital sites such as the eye, skin and lymph nodes were also examined. Sections were cut at 4–5 $\mu$ and stained with haematoxylin and eosin, Gordon and Sweet's silver method for reticulin, PTAH and PAS. From 2 tumours, sections were immediately placed in 2% glutaraldehyde in Sorenson's phosphate buffer at pH 7·4. After post-fixation in osmium tetroxide, the specimens were carried through alcohols and finally embedded in maraglas. 0·5 to 1 $\mu$ sections were cut with a Cambridge Huxley ultra-microtome using glass knives, and stained with toluidine blue, then examined by light microscopy. For nuclear sexing, tissues were stained with thionine and 100 or 200 cells were scored from different parts of the tumour.

In (2) material from the primary genital site was taken from 11 animals. In 7 of these, exudate from the surface of the tumour was removed and allowed to drip into Hank's balanced salt solution (HBSS) containing Colcemid (Ciba) 1 : 6,000,000 and left at room temperature for 1½ hours. In all 11 animals, pieces of fresh tumour were placed in HBSS, minced and Colcemid added for 1½ hours. Because of practical problems imposed by the nature of the veterinary service, it was not always possible to expose the tumour immediately in Colcemid and in fact cases 32, 34, and 35 were left 24 hours. For this reason the results for the tumour were not as good as those for the exudate. After exposure to Colcemid, both exudate and tumour were treated identically. The suspension was spun down and the cells were exposed to hypotonic HBSS for 20 minutes at 37° C. The cells were fixed with methanol : acetic acid, 3 : 1, and flame dried smears stained with Giemsa. Visual counts and analyses were made of as many metaphase cells as possible in all the specimens and suitable cells were photographed, analysed and karyotyped.

### RESULTS

*Pathological observations*

The most striking feature of the tumours was the uniformity of appearance. They were mainly superficially located, occupying the sub-epithelial connective tissue of the penis or vagina in most cases. Secondary compression and atrophy with ulceration of the overlying surface was produced in a few. Most tumours were well circumscribed deeply and laterally and sometimes separated from the adjacent connective tissue by a zone of reactive fibrosis and lymphocyte proliferation. Secondary infection was present in several of the genital tumours.

There were 2 distinct cell types (Fig. 1a). The first, a large uniform rounded pale-staining cell with amphophilic cytoplasm in H. & E. sections and with an eccentric nucleus. This contained a distinct granular chromatin pattern and there were pronounced eosinophilic nucleoli. The cells lay closely packed together but were devoid of intercellular bridges. Cell margins were indistinct in H & E but outlined by PTAH and PAS. These cells made up the bulk of the tumour. Mitoses were conspicuous. Silver impregnation showed the reticulin fibres arranged in thin strands around groups of tumour cells (Fig. 1b). The second cell type was much smaller and more variable. They were small rounded or oval cells with scanty cytoplasm and deeply basophilic nuclei. They were present in clusters of variable size among the tumour sheets and around blood vessels. Most appeared to be lymphoid cells and mitoses were not seen among them.

*Sex chromatin*.—While it is well recognised that the study of sex chromatin in formalin fixed tissues is often unsatisfactory and may give variable results, it was felt to be worthwhile to examine this as a pilot study and to compare the results with those of the later examinations. The percentage of sex chromatin seen in the local skin or epithelium of affected females ranged from 24% to 41%. In the tumour no sex chromatin body was observed in any of the small dark cells. In the larger clear cells, one or more nuclear condensations were seen on the nuclear membrane in a low proportion of cells varying from 1 to 12%. Mostly these did not correspond in size or shape with the normal Barr body. There was no correlation of occurrence or frequency of these chromocentres with the sex of the animal. It was concluded that these more resembled the chromatin condensations frequently seen on the nuclear membrane in malignant cells.

*Cytological observations on air-dried smears of exudate and tumour*

Secondary infection, as judged by the presence of pus cells, was variable. Otherwise 2 distinct types of cell were again seen, the small dark dense cell and the clear cell with prominent chromatin (Fig. 2). The ratio of dark to clear cells in both exudate and tumour and the frequency of " sex-chromatin like " bodies in the 2 types of cell are shown in Table I. In animals 32, 34 and 35 the cells

TABLE I.—*Frequency of Sex Chromatin in the Cells of Exudate and Tumour*

| | | Exudate | | | Tumour | | |
|---|---|---|---|---|---|---|---|
| | | Ratio of dark to clear cells | % Sex chromatin | | Ratio of dark to clear cells | % Sex chromatin | |
| Index no. of dog | Sex | | Dark | Clear | | Dark | Clear |
| 22 | F | 187/13 | 0 | 8 | 94/106 | 0 | 14 |
| 23 | M | 285/15 | 0 | 6 | 17/83 | 0 | 13 |
| 24 | F | 187/13 | 0 | 10 | 95/123 | 0 | 14 |
| 25 | M | 275/25 | 0 | 7 | 27/173 | 0 | 20 |
| 26 | M | 186/14 | 0 | 16 | 4/96 | 0 | 19 |
| 27 | M | 80/20 | 0 | 18 | 50/150 | 0 | 20 |
| 28 | M | 93/7 | 0 | 18 | 13/88 | 0 | 20 |
| 29 | M | 190/11 | 0 | 12 | 42/58 | 0 | 21 |
| 32 | F | — | — | — | 24/76 | 0 | 30 |
| 34 | F | — | — | — | 25/75 | 0 | 22 |
| 35 | M | — | — | — | 70/30 | 0 | 18 |

in the exudate showed considerable degeneration, so no observations were made. Dark cells predominated in the exudate in all cases, whereas in the tumour, the clear cells were predominant in all cases except 35. As in the fixed tissue, no sex chromatin was seen in the dark cells. In the clear cells, several chromocentres were observed in many cells (Fig. 2). Also, in a varying proportion of cells ranging from 6% to 30%, a distinct triangular, slightly refractile body was seen. Its position varied from a central site to the nuclear membrane (Fig. 2). A positive scoring for sex chromatin was only made if this body was on or immediately adjacent to the nuclear membrane. This body was seen in all animals and its incidence was independent of the sex of the animal.

*Cytogenetic observations*

Results from 9 dogs are recorded in Table II. In dog 27, no suitable metaphases were detected and in dog 34 only one, with 59 chromosomes, was seen. In dogs 22 to 29 only the exudate was fruitful, while in 32 and 35 cells from the tumour were analysed.

TABLE II.—*Chromosome Number in the Different Tumours of Nine Dogs*

| | Number of cells | | | | | | | | | |
|---|---|---|---|---|---|---|---|---|---|---|
| Index no. of dog | 22 | 23 | 24 | 25 | 26 | 28 | 29 | 32 | 35 | Total |
| Chromosome number | | | | | | | | | | |
| 36 . | — | — | — | 1 | — | — | — | — | — | 1 |
| 39 . | — | — | 1 | — | — | — | — | — | — | 1 |
| 54 . | — | — | — | 2 | — | — | 2 | 1 | — | 5 |
| 55 . | 1 | 1 | — | 1 | — | 1 | — | 2 | — | 6 |
| 56 . | 1 | 4 | — | — | 2 | 2 | — | 2 | — | 11 |
| 57 . | 2 | — | — | 1 | 4 | 1 | 4 | — | — | 12 |
| 58 . | 3 | 5 | 4 | — | 7 | 3 | 10 | 2 | — | 34 |
| 59 . | 5 | 14 | 5 | 3 | 27 | 17 | 28 | 7 | — | 106 |
| 60 . | 3 | — | 1 | — | 5 | 5 | 2 | 5 | — | 21 |
| 61 . | — | — | — | — | 1 | 1 | 3 | — | — | 5 |
| 62 . | 1 | — | 1 | — | — | — | — | — | — | 2 |
| 64 . | — | — | — | — | — | — | 1 | — | — | 1 |
| 72 . | — | — | — | — | 1 | — | — | — | — | 1 |
| 75 . | — | — | — | — | 1 | — | — | — | — | 1 |
| 77 . | — | — | — | — | 1 | — | — | — | — | 1 |
| 78 . | — | — | — | — | 1 | — | — | — | 1 | 2 |
| 104 . | — | — | — | — | — | 1 | — | — | — | 1 |
| Tetraploid . | — | — | — | — | — | — | — | 2 | 34 | 36 |
| Totals . | 17 | 24 | 12 | 8 | 50 | 31 | 50 | 21 | 35* | 248 |
| Sex . | F | M | F | M | M | M | M | F | F | M |

* Most cells not analysable.

In all tumours except in dog 35, the modal number was 59, with 17% of cells showing 58 and 11% 60 chromosomes. Cells with the normal canine number (78) were seen once in each of 4 of the tumours (Fig. 3). In dog 35, all the cells seen in both tumour and exudate were probably tetraploid. In the 7 analysable cells, the modal number was around 118 and all the other cells seen, except one with the normal canine number, had a similar appearance.

The karyotype in different animals varied only slightly (Table III). There were between 15 and 17 metacentric or submetacentric chromosomes and the rest were acrocentric. In all cases there were 2 consistent marker chromosomes, a long submetacentric (Fig. 4) and a very long acrocentric which had a tendency to a secondary constriction halfway along the long arm. There were also 2 less consistent markers, a large metacentric and a long submetacentric, which sometimes showed negative heteropyknosis. There were usually 4 distinct pairs of metacentrics. The rest were arranged in descending order of size. The sex chromosomes which normally stand out as the only metacentric chromosomes in the canine karotype, were not definitely identified. One small metacentric which could have been a Y chromosome was seen in all cases.

### DISCUSSION

There are several points of interest arising out of the pathological and cytological observations. The pathological appearances and natural history of this tumour separate it from the malignant lymphomas among which this tumour was often classified some years ago. There appears to be 2 distinct cellular elements in this tumour, the clear tumour cell and the dark dense cell. We have concluded

TABLE III.—*The Variation in Karyotype in the Nine Tumours*

| Index no. of dog | Sex | No. of karyotypes | Number of Metacentrics: Markers | Paired | Unpaired | Total | Acrocentrics: Marker | Total | Chromosome number |
|---|---|---|---|---|---|---|---|---|---|
| 22 | F | 3 | 2 SM. 1 M. | 8 | 7 | 15 | 1 | 44 | 57 to 59 |
| 23 | M | 4 | 1 long SM. 1 with neg. hetero-pyknosis | 8 or 10 | 9 or 7 | 17 | 1 | 42 | 59 |
| 24 | F | 3 | 1 SM. 1 M in 2 cells | 6 | 8 | 14–15 | 1 | — | — |
| 25 | M | 2 | 1 SM. 1 M. | 6 | 9 | 15 | 1 | 44 | 59 |
| 26 | M | 5 | 1 SM. 1 M. | 8 or 10 | 8 or 6 | 16 | 1 | 43 | 59 |
| 28 | M | 3 | 2 SM. | 8 | 7, 9 | 15, 17, 34 | 1 variable | 61 | 59, 104 |
| 29 | M | 4 | 3 & 2 | 8 | 7–9 | 15–17 | 1 | 42–44 | 59 |
| 32 | F | 3 | 1 SM. | 8 or 10 | 7 | 15–17 | 1 | 42–44 | 59 |
| 35* | M | 5 | 2 identical long SM. | ? | ? | 26–29 | 2 identical long | 82–90 approx. | 111–119 approx. |

* The quality of these cells was poor, so an exact definition of morphology was not possible. M = Metacentric SM = submetacentric.

that the latter is probably a lymphocyte or lymphoid cell. This appears to be more frequent in the exudate but varies considerably from case to case in the tumour. The reason for the high frequency in the exudate is probably the presence of secondary infection and also the natural longevity of the lymphocyte compared with tumour cells. This might be enhanced by the possible loss of tumour cells during the hypotonic and subsequent treatment. For example, in cases 34 and 35 where no well preserved tumour cells were seen in the exudate, many well preserved lymphocytes were present and the one normal canine mitosis was probably of lymphocytic origin. This observation is borne out by Prier (1966) who found that tumour cells with the 59 chromosome karyotype had almost disappeared from tissue culture after 72 hours, leaving only normal diploid canine cells. It is also interesting to note that the tumour with the highest proportion of lymphoid cells was the one with the apparent tetraploid number.

Because of the inadequate history and follow-up of the animals, it would be very difficult to equate the nature and virulence of the individual tumour with the extent of the lymphoid element. This would seem to be of some importance as the degree of lymphoid reaction may well represent the animal's resistance to the tumour. Several human tumours with a marked lymphoid element are thought to be more benign than similar tumours with none, such as carcinoma of the breast and the salivary gland lympho-epithelioma.

---

### EXPLANATION OF PLATES

FIG. 1a.—Section of tumour, 1 $\mu$ thick, maraglas embedded, Methylene blue. $\times$ 260.

FIG. 1b.—Section of tumour stained with Gordon and Sweet's method for reticulin. $\times$ 260.

FIG. 2.—Cytological smear of tumour showing lymphoid and tumour nuclei, Giemsa. $\times$ 70.

FIG. 3.—Karyotype of normal male canine cell.

FIG. 4.—The karyotype in a typical tumour cell with 59 chromosomes.

1a 1b

2

Thorburn, Gywnn, Ragbeer and Lee.

3

4

The cytological findings are of interest in relation to the nature and aetiology of the tumour. There does not appear to be a definite sex chromatin body. We feel that the triangular body seen in many nuclei in a peripheral location is the nucleolus.

Karlson and Mann (1952) observed a nucleolus in many CVT cells. In our cases there was no relation between the sex of the animal and the presence of the body or any other chromocentre on the nuclear membrane. It is interesting to speculate why there is no apparent sex chromatin. Most benign and many malignant tumours that have been investigated in man show the same nuclear sex as that of the host, though the sex chromatin level may be lower in more anaplastic tumours (Tavares, 1966).

The possibilities are that:

(a) the tumour is male,
(b) it has lost its sex chromatin in the female animal, or
(c) it never had any.

If it is a " male tumour " then presumably it arose originally in a male animal. If it is considered that the sex chromatin is lost, presumably this may be due to the same agent which caused the profound change in the karyotype. The possibility remains that it is not a tumour arising in the dog's tissues but a parasitic collection of cells, in which case the normal processes of X inactivation which occur initially in early embryonic life in mammals are either by-passed or do not occur.

A similar situation probably exists in the contagious reticulum cell sarcoma of the Syrian hamster. Cooper *et al.* (1964) could demonstrate only an XY complement in the tumours implanted in females though they did not examine for sex chromatin.

The cytogenetic findings are essentially similar to those reported from Japan (Makino, 1963), the United States (Weber *et al.*, 1965) and France (Barski and Cornefert-Jensen, 1966). The number and distribution of chromosomes differ only slightly, there being a modal number of 59 with 15 to 17 metacentrics. Some of the individual chromosomes bear a striking resemblance to those in the above reports. There is an almost identical long submetacentric marker in all reports and in many of our tumour cells and those of Makino there is a similar large metacentric.

One difference seen in our cases, was the presence of a single large acrocentric marker which had a marked tendency to constriction halfway along the long arm.

The karyotype did not permit identification of the sex of the tumour. These findings are of considerable interest in the light of the isolation of Jamaica in the canine world. The importation of dogs has only been allowed for the last 25 years *via* the United Kingdom, which imposes a strict 6-month quarantine on dogs.

In addition, any dogs admitted are likely to have been well kept and free of tumour as the CVT is uncommon in the United States and Canada and rare in Britain. The likelihood of introduction from another country in the last 25 years is extremely small.

The main points of interest in the CVT are: (a) whether the CVT is a neoplasm, and (b) whether it is caused by a virus.

Although the active and anaplastic appearance of the CVT is much in favour of it being a malignant neoplasm, and the numerous names given it in the past reflect this aspect, its behaviour is not like that of a malignant tumour. Many do not recur after simple diathermy treatment. The tumours appear to occur more frequently in young animals. In our experience they uncommonly spread beyond the local lymph nodes. All these facts suggest that the dog develops increasing immunity to the tumour. The development of immunity or resistance was shown clinically by Karlson and Mann (1952) who could not reintroduce the tumour in dogs already affected, and is suggested also by a rise in gamma globulin levels in dogs with experimentally induced tumours (Adams and Chineme, 1967). In those animals that have been seen with tumour at extra-genital sites, e.g. the eye or the skin, there is a possibility that direct inoculation into the conjunctive or a cut had occurred. However, we have not performed cytogenetic examination on any extra-genital tumours and although the histological appearances are identical to the genital tumours, the possibility remains that they did not have the identical karyotype.

Further immunological interest would be provided by studying the effect of anti-lymphocyte serum on the CVT.

Previous work has been equivocal, Carteaud (1965) quotes Jean *et al.* and Thiery as having produced completely different responses, the former getting regression of the tumour and the latter obtaining enhancement of tumour growth. The latter is what one might expect if the extensive lymphoid component of the tumour has any significance.

Much work has been done to prove the viral origin of the CVT, though attempts to induce it with filtrable material have been unsuccessful. The Italian workers, Dozza and Torlone (1960) and Ajello and Gimbo (1965) have reported success in inducing the tumour using nuclear and cytoplasmic fractions produced by trypsinisation and centrifugation respectively.

However, these observations do not prove a viral aetiology, as it has been shown that chromosomes of mouse leukaemia cells can be incorporated into mouse macrophages *in vitro* (Chorazy *et al.*, 1963) though whether they remain intact after 26 hours was not demonstrated. The absence of nuclear sex correlation would be against a viral aetiology, as one would expect that neoplastic change induced in each new host by a virus would give a tumour showing the same sex chromatin pattern, and is more in favour of direct inoculation of tumour cells.

Also, the vast and consistent difference between the chromosome constitution of the CVT and of the dog is against virus induction of each new tumour. It is only paralleled in spontaneous and experimentally induced tumours by the reticulum cell sarcoma of the Syrian hamster (Cooper *et al.*, 1964) which has a similar constant karyotype widely different from the hamster's normal constitution.

Although the change in karyotype from a 78 chromosome mode with acrocentric autosomes to 59 with numerous metacentrics could be explained by centric fusion of many chromosomes, it is unlikely that a single oncogenic agent could induce such a profound and consistent change in each new animal. Possibly this tumour, whose history dates back at least to 1876, was originally produced by a virus and then developed its stable karyotype which has the property of conferring on the cells a remarkable capacity for avoiding transplant rejection. It has little deleterious effect on the host and its behaviour in general resembles more that of a parasite than a tumour.

### SUMMARY

Cytological and cytogenetic observations are recorded on Jamaican dogs with spontaneous transmissible venereal tumour. Fixed tissue from 22 dogs was examined for pathological study and sex chromatin, and fresh tissue and exudate from the tumour from 9 dogs were examined for cytological features, and chromosome studies.

The pathological appearances exclude this tumour from the lymphoma group and show 2 distinct cells, the tumour cell and a lymphoid element. These 2 cell types are seen in the exudate and fresh tissue. The lymphoid cells show no sex chromatin body. The tumour cells show a body which occurs independently of the sex of the animal in 10–30% of cells and is thought to be a nucleolus. The chromosome number and karyotype is 58 or 59 with a similar pattern to that seen in the United States and Japan. The aetiology is discussed in the light of these findings and the relative isolation of Jamaica in the canine world.

The cytogenetic work was supported by grants from the Wellcome Trust and the Standing Advisory Committee for Medical Research in the Caribbean.

### REFERENCES

Adams, E. W. and Chineme, C. M.—(1967) *Cornell Vet.*, **57**, 572.

Ajello, P. and Gimbo, A.—(1965) *Archo vet. ital.*, **18**, 275.

Barski, G. and Cornefert-Jensen, Fr.—(1966) *J. natn. Cancer Inst.*, **37**, 787.

Carteaud, A. J. P.—(1965) In ‘ Comparative Physiology and Pathology of the Skin ’. Edited by Rook, A. J. and Walton, G. S. Oxford (Blackwells), p. 685.

Chorazy, M., Bendich, A., Borenfreund, E. and Hutchison, D. J.—(1963) *J. Cell Biol.*, **19**, 59.

Cooper, H. L., Mackay, C. M. and Banfield, W. G.—(1964) *J. natn. Cancer Inst.*, **33**, 691.

Dozza, G. and Torlone, V.—(1960) *Veterinaria ital.*, **11**, 647.

Higgins, D. A.—(1966) *Vet. Rec.*, **79**, 67.

Karlson, A. C. and Mann, F. C.—(1952) *Ann. N.Y. Acad. Sci.*, **54**, 1197.

Makino, S.—(1963) *Ann. N.Y. Acad. Sci.*, **108**, 1106.

Prier, J. E.—(1966) *Nature, Lond.*, **212**, 724.

Stewart, H. L., Snell, K. C., Dunham, L. J. and Schlyen, S. M.—(1959) In ‘ Transplantable and Transmissible Venereal Tumours of Animals ’. Washington, D.C. (Armed Forces Institute of Pathology) p. 364.

Tavares, A. S.—(1966) In ‘ The Sex Chromatin ’. Edited by Moore, K. L. Philadelphia and London (W. B. Saunders), p. 410.

Weber, W. T., Nowell, P. C. and Hare, W. C. D.—(1965) *J. natn. Cancer Inst.*, **35**, 537.